# Gastroprotective activity of *Hypericum perforatum* extract in ethanol-induced gastric mucosal injury in Wistar rats: A possible involvement of H+/K+ ATPase α inhibition

**DOI:** 10.1016/j.heliyon.2020.e05249

**Published:** 2020-10-16

**Authors:** Sargul H. Sofi, Sheila M. Nuraddin, Zahra A. Amin, Hazem A. Al-Bustany, Marwan Q. Nadir

**Affiliations:** a Pharmacognsy Department, College of Pharmacy, Hawler Medical University, Erbil, Iraq; b Basic Science Department, College of Medicine, Hawler Medical University, Erbil, Iraq

**Keywords:** *Hypericum perforatum*, Gastric ulcer, H+/K+ ATPase α, AutoDock, Vina, Agricultural science, Biological sciences, Immunology, Veterinary medicine, Health sciences

## Abstract

*Hypericum perforatum* (HP) is a plant native to Asia and Europe. It has been documented to enclose medical effects against many disorders such as anxiety, depression and burns. This experiment was performed to evaluate the gastro-protective effect of *Hypericum perforatum* leaf extract in ethanol induced gastric ulcer in rats as compared to esomeprazole (the drug of choice for stomach ulcers). The mechanism of action was performed by Auto Dock Vina method.

Ethanol ingestion up regulated the inflammatory reaction as demonstrated by rise of gastric proinflammatory TNF-α with a decline of IL-1β. On the other hand, the phytochemical screening of HP revealed the presence of alkaloids, flavonoids, tannins, phenols, steroids and saponins. The high dose of HP group shows mild injuries to the gastric mucosa which is comparable to the esomeprazole group, in contrast, severe damages are observed in the gastric mucosa of the ulcer control rats group. I*n silico* results revealed that Amentoflavone and Quercitrin have highest affinity and very good interactions with H+/K+ ATPase α active site. This study showed that HP is nearly as effective as esomeprazole to prevent ethanol induced gastric ulcer the plant extract and it has more binding affinity than esomeprazole to gastric proton pumps.

## 1 Introduction

Gastric ulcer disease is an illness which affects large number of people worldwide. It will develop when there is an imbalance between the protective factors like (bicarbonate, mucus layer, mucosal blood flow), and aggressive factors like (*Helicobacter pylori*, HCl, pepsins, non-steroidal anti-inflammatory drugs (NSAIDs), bile acids, ischemia, hypoxia, smoking and alcohol) at the luminal surface of the epithelial cells (Awaad et al., 2013). Gastric ulcers are caused either by using NSAIDs or infection with *H. pylori*, NSAIDs works via inhibition of COX enzymes and thus inhibition of the production of prostaglandins which is a gastro-intestinal cell protective agent (Papadakis and McPhee, 2019). There are many treatments options for peptic ulcer like (proton pump inhibitors, histamine-2 receptor blockers, prostaglandin analogue, misoprostol, bismuth sucralfate, and antibiotics for eradication of *H. pylori*, But treatment of peptic ulcer becomes difficult with these drugs because of unpredictable adverse effects of these drugs when used for a long term (Cadirci et al., 2007).

Ethanol which is a colorless volatile liquid is an injurious agent, it is associated with many pathologies and it has been given orally to experimental animals to cause ulcers and gastric lesions. Ethanol products a distraction in the integrity of the gastric barrier through exfoliation of cells, and increasing mucosal permeability and in some cases cause bleeding, The flowing of neutrophils to the site of damage stimulate elevated concentrations of reactive oxygen species (ROS) also other mediators of inflammation, causing oxidative damage with harmful effects on cells. Oxidative stress has a role in ethanol-induced gastric mucosal injury (Khazaei and Salehi, 2006; Sibilia et al., 2003).

*Hypericum perforatum* a plant from family (Hypericaceae) was used as a medical agent to treat some conditions, especially as a ‘nerve tonic’ and in the treatment of Central Nervous system disorders. It can be used to treat mild and moderate forms of depression and is registered in the UK for the treatment of ‘slightly low mood and mild anxiety’. Herbal products that contain *Hypericum perforatum* have been among the most-selling herbal products in developed countries in recent years. The dried herb (consisting mainly of the flowering tops, including leaves, unopened buds and flowers) is the part used pharmaceutically (Heinrich et al., 2017). It has many actions such as antidepressant, antiviral, anxiolytic and antibacterial activity. *Hypericum perforatum* contains a number of naphthodianthrones, which include hypericin and pseudohypericin, and the prenylated phloroglucinols, such as hyperforin and adhyperforin. Initially, hypericin is consider to be the antidepressant constituent of *Hypericum perforatum*, although evidence has now appeared that hyperforin is also a major constituent require for antidepressant activity. *Hypericum perforatum* also contains some biologically active constituents, such as flavonoids. The leaves and flowers also contain an essential oil, of which the major components are b-caryophyllene, caryophyllene oxide spathulenol, tetradecanol, viridiflorol, a- and b-pinene, and a- and b-selinene (Heinrich et al., 2017).

The aim of the current experiment was screen the phytochemical content of *Hypericum perforatum* and to study the antibacterial activity beside evaluating the gastroprotective effect on ethanol induced gastric ulcer in a rat model and to test the mechanism of action by *insilico* Autodock Vina method.

## 2 Materials and methods

### 2.1 Preparation of ethanolic plant extract

The leaf of *Hypericum perforatum* was dried in a hot air oven at 40–50 C for a week. The dried plant material was powdered using mixer grinder, and subjected to ultrasonic bath extractor with 80% ethanol for 3 h at 40 °C. The mixture was evaporated to dryness in a rotary evaporator and stored in refrigerator. The condensed extract was used for phytochemical screening.

### 2.2 Phytochemical screening procedures

The leaf of the plant was tested for secondary metabolites according to standard procedures (Maobe et al., 2013) (Tiwari et al., 2011).

#### 2.2.1 Keller-killiani test for cardio active glycosides

Extract (0.5gm) was dissolved in 3 ml glacial acetic, 2 drops of (1%) ferric chloride were added, 1 ml of concentrated sulphuric acid (H_2_SO_4_) was added slowly and the result was recorded.

#### 2.2.2 Foam test for saponins

Dried extract (0.5gm) dissolved in 10 ml distilled water and transfer the extract into test tube. Then they were stoppered and shaken dynamically for about 30 s. The test tubes were stand in a vertical site and detected over a 10-min and the result was recorded.

#### 2.2.3 Ferric chloride test for tannins and phenols

Few drops of 1% ferric chloride reagent were added to l ml of extract and the result was recorded.

#### 2.2.4 Molisch reagent test for carbohydrate

Ten gm of α-naphthol was dissolved in 100 ml ethanol 95%, 2–3ml of extract was added, and then 2 drops of molisch reagent was added into test tube and mixed well by shaking. Added about 3 ml conc. H_2_SO_4_ down the side of test tube to form the layer below the sugar solution and result was recorded.

#### 2.2.5 Fehling test for carbohydrates

Add to 5ml of extract, 10ml of fehling solution (5ml of fehling A and 5ml of fehling B) and heat to boiling. Developed of red color precipitate indicated the presence of reducing sugar.

#### 2.2.6 Libermann-Burchard test for unsaturated sterols and or triterpenes

One gm of crude extract in 10 ml chloroform was dissolved, filtered and added to the filtrate 1 ml of acetic acid anhydride, followed by 2 ml of sulphuric acid down the wall of the test tube and result was recorded.

#### 2.2.7 Sodium hydroxide test for flavonoids

Two ml of NaOH (5%) was added to the extract and the color change noted, followed by addition of diluted HCl (5%) and the color change was recorded.

#### 2.2.8 Dragendorff reagent test for alkaloids

Two ml of diluted sodium hydroxide (5% NaOH) solution added to the extract; extraction is then carried out with organic solvent (chloroform). Aqueous acid 1–2 ml (5% HCl) was added to the organic liquid in a separatory funnel and allowed to separate; the aqueous extract is used for detection of alkaloidal compounds by adding few drops of dragendorff reagent and the result was recorded.

#### 2.2.9 Wagner's reagent for alkaloids

(solution of iodine in potassium iodide).

#### 2.2.10 Borntrager's test

2 ml of an extract with 1–2 drops of ammonia in a test tube, the appearance of a rose-pink to cherry red color confirms its presence.

### 2.3 Antibacterial procedure

#### 2.3.1 Antibacterial activity assay

Kirby – Bauer assay was used for antibacterial activity. Different concentrations of plant extract were impregnated in blank discs (200-25 mg/ml).The bacteria were cultured on MHA dishes at 37 °C then growing cultures were adjusted to 0.5 Mc Farland. For the determination of the antibacterial activity, the diameters of the inhibition zones around the discs were measured after 24 h. Ceftazidime/clavulanic acid 30/10 mcg/disc was used as the controls of the study. The whole assays were repeated thrice and the mean values were set.

#### 2.3.2 Antibiofilm activity

The anti-biofilm activity of *Hypericum perforatum* was studied by a micro plate assay. Trypticase Soy Broth (TSB) enhanced with 5% D-glucose was used as the culture media. Thoroughly 200 μL of bacterial suspension was mixed with 20μL *Hypericum perforatum* extract (100 mg/mL), then incubated at 37 °C for 48 h. Subsequently the microplate wells were washed with sterilized distilled water two times in order to eliminate the planktonic bacterial growth. Then the wells were stained for 10 min with 0.1% crystal violet dye. The wells were washed one more time to eliminate the excess dye solution. Finally, eluted for 30 min with 150 μl 95% ethanol, optical density (OD) of the solutions was measured at 630 nm.

The antibiofilm activity of *Hypericum perforatum* extract against *Escherichia coli* and *Staphylococcus aureus* bacteria were calculated with the following equation:

% Antibiofilm activity = [(ControlOD−SampleOD)/ControlOD]×100,

whereas the bacterial cell suspensions without *Hypericum perforatum* was considered as the control of the assay.

### 2.4 Preparation of H+/K+ ATPase α protein and plant extracts

Crystal structures of H+/K+ ATPase α (gastric proton pumps) downloaded from Swiss-Prot Database (https://swissmodel.expasy.org) (ID: P20648) (Elshamy et al., 2020) (Yan et al., 2004). Protein structures preparation for molecular docking accomplished by Discovery Studio 4.1 (http://accerys.com) (Biovia, 2017), and Molecular Graphics Laboratory (MGL) Tools 1.5.6 (http://mgltools.scripps.edu) (Bibi et al., 2019) used to add polar hydrogens and saved in (pdbqt) file format. In present study, all available 9 plant extracts from *Hypericum perforatum* leaf (Table 1) were investigated for targeting H+/K+ ATPase α. The 2D structures (Figure 1) drown using ChemDraw Pro (www.cambridgesoft.com) (Z. Li, Wan, Shi and Ouyang, 2004), then converted to 3D structures in (.pdb) file format using Discovery Studio 4.1 (http://accerys.com) (Biovia, 2017), and finally converted to (.pdbqt) which required to feed AutoDock Vina using Open Babel graphical user interface (http://openbabel.org/) (O'Boyle et al., 2011).

**Table 1:** List of structures.

#	Compound Name	References
1	Inhibitor	(Elshamy et al., 2020; Elshamy et al., 2020)
2	Adhyperforin	(Nahrstedt and Butterweck, 1997; Nahrstedt and Butterweck, 1997)
3	Amentoflavone	(Saddiqe et al., 2010; Saddiqe et al., 2010)
4	Hyperforin	(Nahrstedt and Butterweck, 1997; Nahrstedt and Butterweck, 1997; Saddiqe et al., 2010; Saddiqe et al., 2010, 2010)
		(Smelcerovic et al., 2006; Smelcerovic et al., 2006)
5	Hypericin	(Nahrstedt and Butterweck, 1997; Nahrstedt and Butterweck, 1997; Saddiqe et al., 2010; Saddiqe et al., 2010, 2010)
		(Tatsis et al., 2007; Tatsis et al., 2007; Smelcerovic et al., 2006; Smelcerovic et al., 2006)
6	Hyperoside (Hyperin)	(Nahrstedt and Butterweck, 1997; Nahrstedt and Butterweck, 1997; Smelcerovic et al., 2006; Smelcerovic et al., 2006, 2006)
7	Pseudohypericin	(Saddiqe et al., 2010; Saddiqe et al., 2010; Tatsis et al., 2007; Tatsis et al., 2007; Smelcerovic et al., 2006; Smelcerovic et al., 2006)
8	Quercetin	(Nahrstedt and Butterweck, 1997; Nahrstedt and Butterweck, 1997; Saddiqe et al., 2010; Saddiqe et al., 2010, 2010)
9	Quercitrin	(Smelcerovic et al., 2006; Smelcerovic et al., 2006)
10	Rutin	(Nahrstedt and Butterweck, 1997; Nahrstedt and Butterweck, 1997; Saddiqe et al., 2010; Saddiqe et al., 2010, 2010)
		(Smelcerovic et al., 2006; Smelcerovic et al., 2006)
11	Esomeprazole (negative control)	(Kendall, 2003; Kendall, 2003)

**Figure 1:**
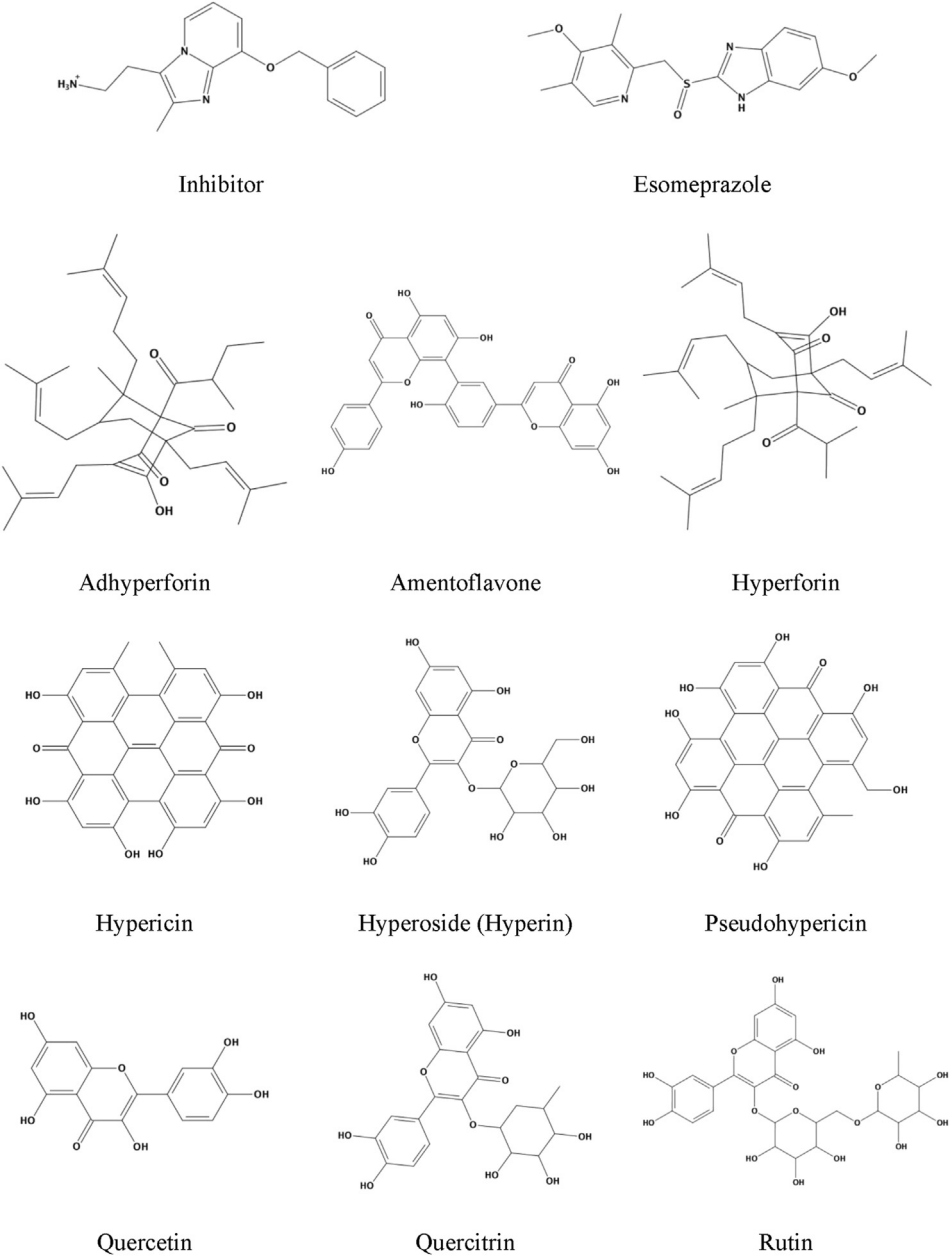
Diagrams showing 2D structures of docked plant extracts.

#### 2.4.1 AutoDock Vina

The docking site for the inhibitor on H+/K+ ATPase α (gastric proton pumps) was defined by forming a grid box that involved manipulating a colored box (x, y, z) covering the active site. In our docking experiments 20 × 20 × 20 Å dimensions were used to cover the binding site with a grid point spacing of 1.0 Å, and center grid boxes was 50.121, -14 and -7 for X, Y and Z dimensions respectively (Trott and Olson, 2010). All required data including receptor, ligand, grid box, coordinates, and exhaustive search, written in a configuration text document file, which was required to feed the AutoDock Vina 1.1.2 (Trott and Olson, 2010). AutoDock Vina was accomplished using Windows 8.1 operating system with 4 CPUs and every compound separately were well docked to H+/K+ ATPase α protein. Six runs were performed for every single compound.

### 2.5 Gastroprotective procedure

All experimental procedures were taken under a protocol approved by the Ethical Committee of College of Pharmacy, Hawler Medical University (Ethics number: HMU.PH.EC, 190720-110) and according to the ARRIVE guidlines (Animal Research: Reporting *In Vivo* Experiments) for reporting animal research.

#### 2.5.1 Experimental animals

The method of the experiment was following the method of Fard et al. (2011) with minor changes. Stomach ulcer was induced by drenching (5 mL/kg) absolute Ethanol. Male Wistar rats weighing (200–240 gm) were used. They were fasted two days prior to the experiment; however they were allowed to drink water until 2 h before the experiment time. Rats were distributed randomly into 4 groups (6 rats each). The experiment dose was 5 mL/kg for all treatment subjects.

1) Ulcer positive group (Group I rats) were gavage with sterilized distilled water.
2) Ulcer negative group (Group II rats) was gavage with 20 mg/kg Esomeprazole.
3) Groups III and groups IV were gavage with 250 and 500 mg/kg of *Hypericum perforatum* extract separately.

After 60 min all rats were gavage absolute ethanol. Then after another 60 min the animals were sacrificed, stomachs were detached and the gastric ulcer lesions were measured.

#### 2.5.2 Ulcer measurements

Gastric ulcer measurements were observed by cutting the stomach along the superior curvature. Gastric content samples were used for the analysis for hydrogen ion concentration by pH-meter titration with 0.1 N NaOH solutions. Stomach mucosa was scraped and the obtained mucus was weighed (Tan et al., 2002). Gastric ulcers were reported as extended bands of hemorrhagic lesions along the axis of the stomach. Dissecting microscope (1.8X) was used for measuring the width (mm) and the length (mm) of the ulcers observed. The ulcer area calculations were measured following the formula's described by (Al Batran et al., 2013).

#### 2.5.3 Analysis of IL-1β and TNFα

On the surgery day, the blood was collected and serum was separated for the cytokines analysis. Rat IL-1β and TNF α Platinum ELISA kits (affymetrix-eBioscience, Vienna, Austria) were used following the manufacturer's instructions.

## 3 Results

### 3.1 Phytochemical screening of the *Hypericum perforatum* extracts

The preliminary phytochemical screening was performed on ethanol extracts of *Hypericum perforatum*, the results indicated the presence of a number of important phytochemical natural product group Table 2.

**Table 2:** *In vitro* qualitative phytochemical analysis of ethanolic extract of leaves of *H perforatum*.

Tests for phytoconstituents	Results	
Cardiac glycosides	**Keller-killiani** Formation of reddish brown junction	+ve
Saponins	**Foam** Foam produced persist for 10 min	+ve
Anthraquinones	**Borntrager's test** The appearance of rose-pink color	+ve
Tannins and phenols	**Ferric chloride** Formation of bluish green color	+ve
Carbohydrates	**Molisch** Formation of violet ring **Fehling test** Red precipitate	+ve
Steroids	**Libermann-Burchard** Reddish brown ring at the interface	+ve
Flavonoids	**Sodium hydroxide** Formation of intense yellow which turn to colorless on the addition of diluted acid	+ve
Alkaloids	**Dragendroff** Red precipitate formation **Wagner's reagent** Reddish-brown precipitate	+ve

(+ve) presence, (-ve) absence of natural product in the plant.

The change of color was observed when the test reagent was added to the prepared sample for the phytochemical test. The result recorded as present (+) or absent (-) depending on the outcome of the test.

### 3.2 Antibacterial activity of *Hypericum perforatum*

Results found that the extract of *Hypericum perforatum* showed positive activity against both Gram-positive and Gram-negative bacteria and the highest plant dose (200 μg/mL) has gave results almost same like the positive antibiotic control Ceftazidime/Clavulanic acid as shown in Table 3. While the anti-biofilm formation results revealed that the plant extract prevented the Gram-negative bacteria *E. coli* to produce biofilm however the Gram-positive bacteria *S. aureus* produced it in a dose dependent manner where decreases when the *Hypericum perforatum* plant extract dose increase (Table 4).

**Table 3:** Antibacterial activity of ethanolic extract of *Hypericum perforatum.*

Concentration (μg/mL)	*S.aureus*	*E. coli*
HP 200	>5	3
HP 100	4	2.4
HP 50	3.3	2
HP 25	3	R
CAC 30/10	>5	3.4

HP: *Hypericum perforatum*, CAC: Ceftazidime/Clavulanic acid, R: Resistant.

**Table 4:** Antibiofilm activity of ethanolic extract of *Hypericum perforatum.*

Concentration (μg/mL)	*S.aureus*	*E. coli*
HP 200	>0.240	N
HP 100	0.24	N
HP 50	<0.120	N
HP 25	<0.120	N

HP: *Hypericum perforatum*, n: not formed.

### 3.3 AutoDock Vina

After applying effective docking protocol, all compounds were successfully docked into H+/K+ ATPase α protein active site. Our results discovered that studied compounds showed different affinities toward the protein, docking analysis demonstrate that among used structures (Table 5); **Amentoflavone, Quercitrin, Hyperoside (Hyperin), Quercetin** have highest affinity and very good interactions with H+/K+ ATPase α active site and gives docking score -10.5, -9.5, -8.7, -8.3 kcal/mol respectively, when compared with inhibitor and esomeprazole (negative control) which gives -7.7, and -8.3 kcal/mol respectively.

**Table 5:** Average docking scores (kcal/mol) for plant extract compounds docked into H+/K+ ATPase α protein.

#	Compound Name	Run 1	Run 2	Run 3	Run 4	Run 5	Run 6	Average
**1**	P20648 Inhibitor	-7.7	-7.7	-7.6	-7.6	-7.7	-7.7	**-7.7**
**2**	Esomeprazole (negative control)	-8.3	-8.2	-8.3	-8.3	-8.3	-8.3	**-8.3**
**3**	**Amentoflavone**	-10.5	-10.6	-10.5	-10.6	-10.5	-10.5	**-10.5**
**4**	**Quercitrin**	-9.5	-9.5	-9.5	-9.5	-9.5	-9.5	**-9.5**
5	**Hyperoside (Hyperin)**	-8.6	-8.6	-8.6	-8.6	-8.6	-8.6	**-8.6**
6	**Quercetin**	-8.3	-8.3	-8.3	-8.3	-8.3	-8.3	**-8.3**
7	Rutin	-7.8	-7.5	-7.4	-7.4	-7.4	-7.4	-7.5
8	Adhyperforin	-3.8	-3.8	-5.0	-4.8	-3.8	-5.1	-4.4
9	Hyperforin	-3.9	-3.9	-4.0	-3.9	-5.2	-3.9	-4.1
10	Hypericin	-3.4	-3.2	-3.2	-3.4	-3.2	-3.2	-3.3
11	Pseudohypericin	-3.3	-3.3	-3.2	-3.3	-3.3	-3.2	-3.3

Bold texts indicate the docking scores (≤ -8.3) of the docking compounds as compared to the Esomeprazole (negative control); i.e. compound no. 3 (Amentoflavone) have the lowest energy (The highest afinity) to the protein.

**Figure 2:**
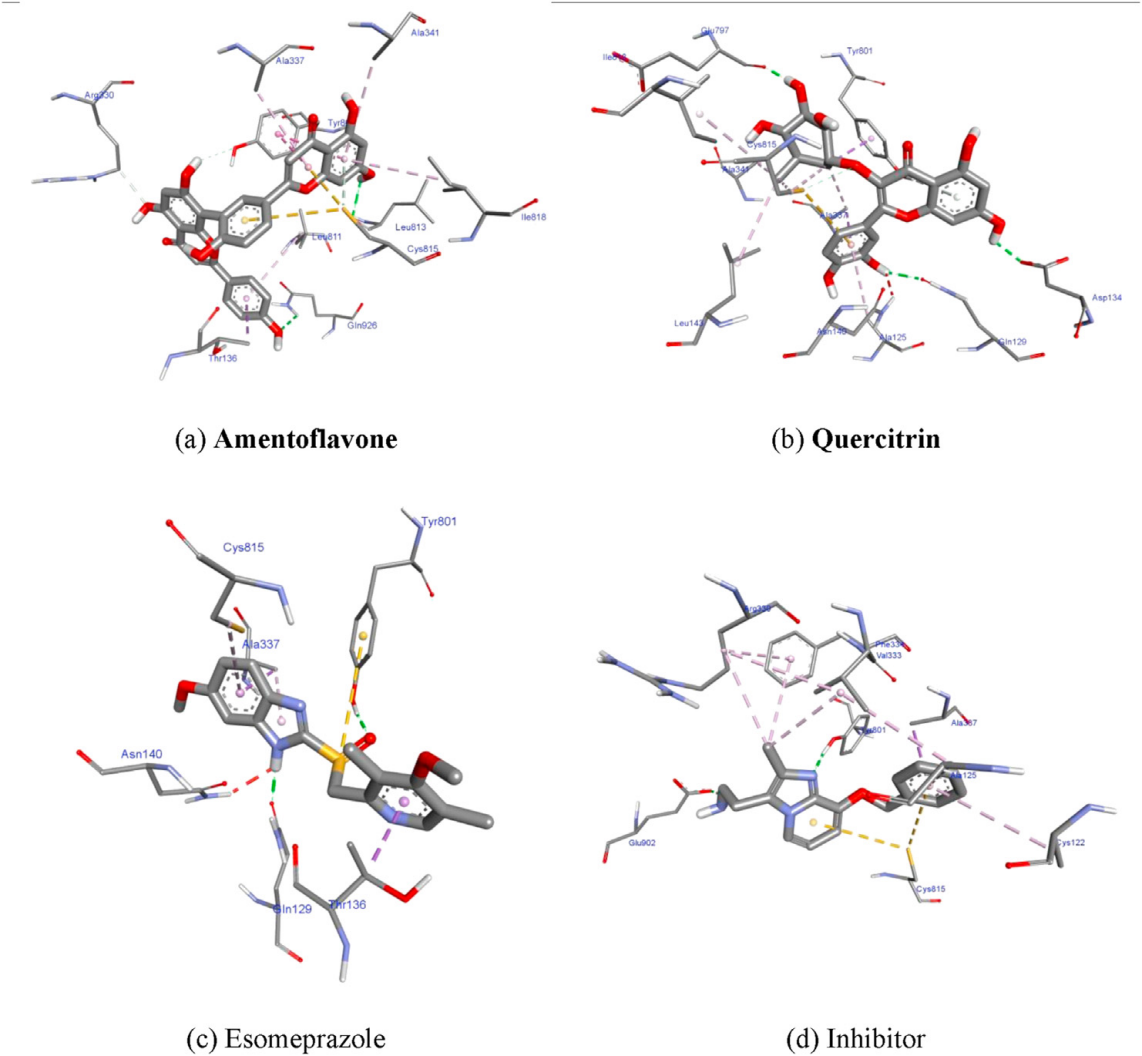
Showing interacted residues of protein with the compounds; (a) Amentoflavone; (b) Quercitrin; (c) Esomeprazole; (d) Inhibitor.

**Amentoflavone** Figure 2 (a) were successfully docked into H+/K+ ATPase α protein which showed highest affinity (lowest energy) -10.5 kcal/mol docking scores. Hydrogen bond and hydrophobic interactions observed and conquered the affinity in binding pockets as well. Hydrogen bond interactions were with ARG^330^, LEU^813^, CYS^815^, GLN^926^, and hydrophobic interactions with THY^136^, ALA^337,341^, TYR^801^, LEU^811^, CYS^815^, ILE^818^.

**Quercitrin** Figure 2 (b) were also formed: hydrogen bond, and hydrophobic interactions giving -9.5 kcal/mol and conquered the affinity of the most favorable binding pockets as well. Hydrogen bond interactions were with GLN^129,797^, ASP^134^, TYR^801^, and hydrophobic interactions with ALA^125,337,341^, ASN^140^, LEU^143^, CYS^815^, and ILE^818^.

**Esomeprazole** (negative control) Figure 2 (c) showed highest affinity docking score when compared to the inhibitor, it formed: Hydrogen bond interactions with residues: GLN^129^, and THY^801^, a hydrophobic interactions THR^136^, ALA^337^, and CYS^815^. Unfavorable non-bonded interactions also observed with ASN140.

**Inhibitor** Figure 2 (d) which showed -7.7 kcal/mol lowest energy as docking score formed: hydrogen bond and hydrophobic interactions observed in the active site. Hydrogen bond interactions were with TYR^801,^ and GLU902, and hydrophobic interactions with CYS^122^, ALA^125, 337^, ARG^330^, VAL^333^, PHE^334^, also pi-sulfur interaction observed with CYS^815^.

### 3.4 Gastroprotective activity

Results showed that rats pre-treated with *Hypericum perforatum* plant extract before being given absolute ethanol had significantly reduced areas of gastric ulcer formation compared to rats pre-treated with only 10%Tween 20 (ulcer control group) (Table 6) which means that *Hypericum perforatum* significantly suppressed the formation of ulcers. The ulcer inhibition percentages were (68%, 95% and 97%) for Esomeprazole, 100 mg/mL *Hypericum perforatum* and 200 mg/mL *Hypericum perforatum* respectively. Also the acidity of gastric content (pH) in experimental animals pretreated with high dose of *Hypericum perforatum* (pH = 2.3 ± 0.7mEq/I) or Esomeprazole (pH = 3.7 ± 1.7mEq/I) was significantly decreased compared with that of the ulcer control group (pH = 5.7 ± 0.7mEq/I) at (p < 0.05).

**Table 6:** Antiulcer activity of *Hypericum perforatum* against ethanol-induced gastric injury.

Animal group	Pre-treatment (5 mL/kg)	Ulcer area (mm2)	Inhibition (%)	Mucus weight (mg)	PH (mEq/l)	P value
Vehicle (Ulcer Positive)	10% Tween 20	220.8 ± 16.8	…	957 ± 13.2	5.7 ± 0.7	….
Control (Ulcer Negative)	20 μg/kg Esomperazole	55.2 ± 2.9∗	68∗	365 ± 19.3∗	3.7 ± 1.7	0.303
High Dose	200 mg/kg plant extract	7.2 ± 0.00∗	97∗	456.33 ± 5.8∗	2.3 ± 0.7	0.009
Low Dose	100 mg/kg plant extract	12 ± 2.4∗	95∗	626.67 ± 11.2	3.3 ± 1.3	>0.0001

∗ The values are expressed as the mean ± SEM. Indicates significance at p < 0.05 compared to the ulcer positive group.

**Figure 3:**
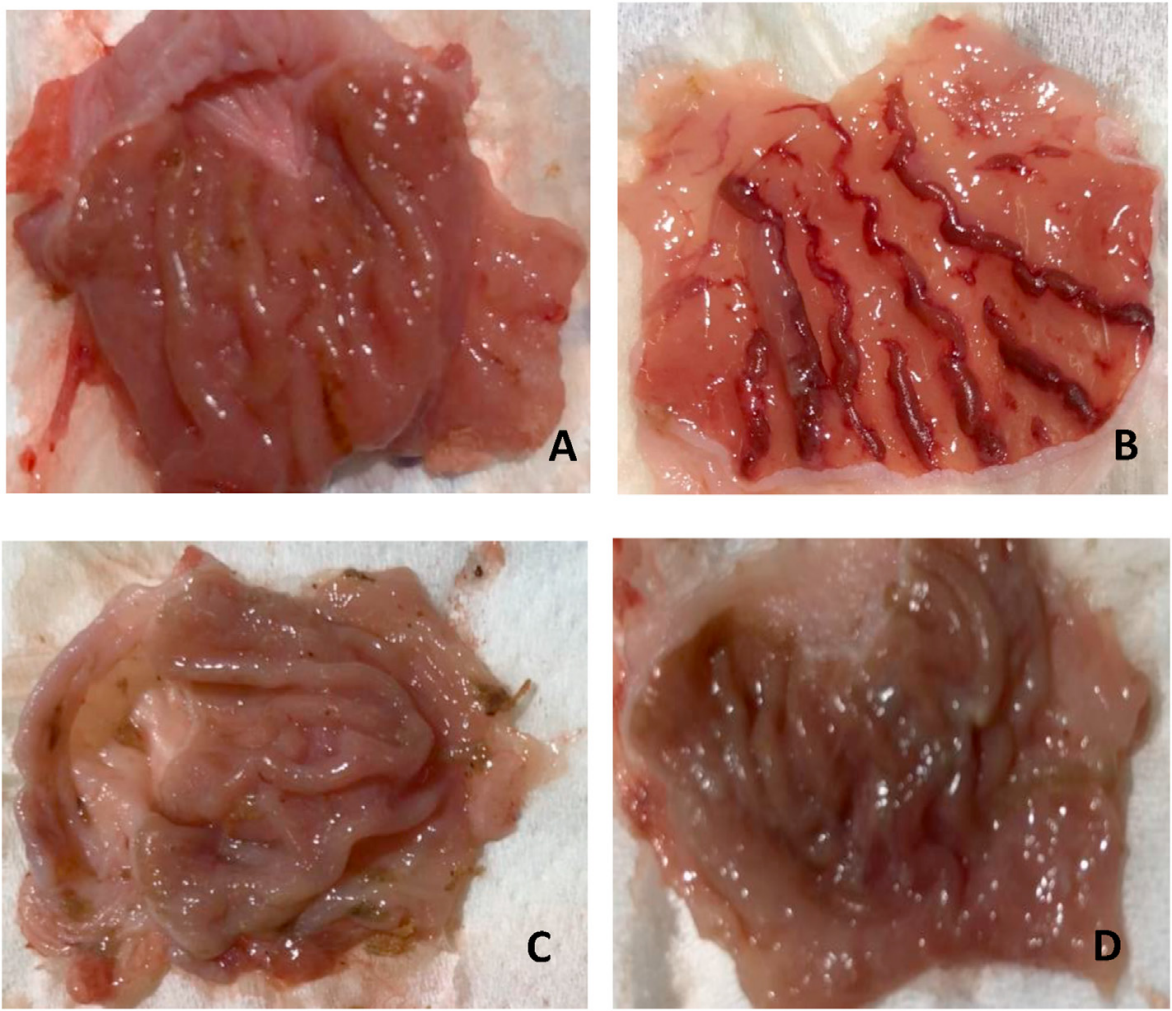
Effect of *Hypericum perforatum* on macroscopic appearance of the gastric mucosa. The esomeprazole (O group) shows no injuries to the gastric mucosa (A). Severe injuries are observed in the gastric mucosa of the ulcer control group (T group) figure (B). The HHD group (200 mg/kg *Hypericum perforatum*) shows mild injuries to the gastric mucosa (C) while HLD group (100 mg/kg *Hypericum perforatum*) shows moderate injuries in the gastric mucosa (D**).**

Moreover it was interesting to note the flattening of gastric mucosal folds in rats pre-treated with *Hypericum perforatum*. Animals pre-treated with 10% Tween 20 showed extensive stomach lesions. Severe hemorrhagic lineage was observed indicating that gastric ulcer was completely formed. Fortunately, Pre-treatment with Esomeprazole and the plant extract of *Hypericum perforatum* reduced the formation of gastric lesions (Figure 3).

**Figure 4:**
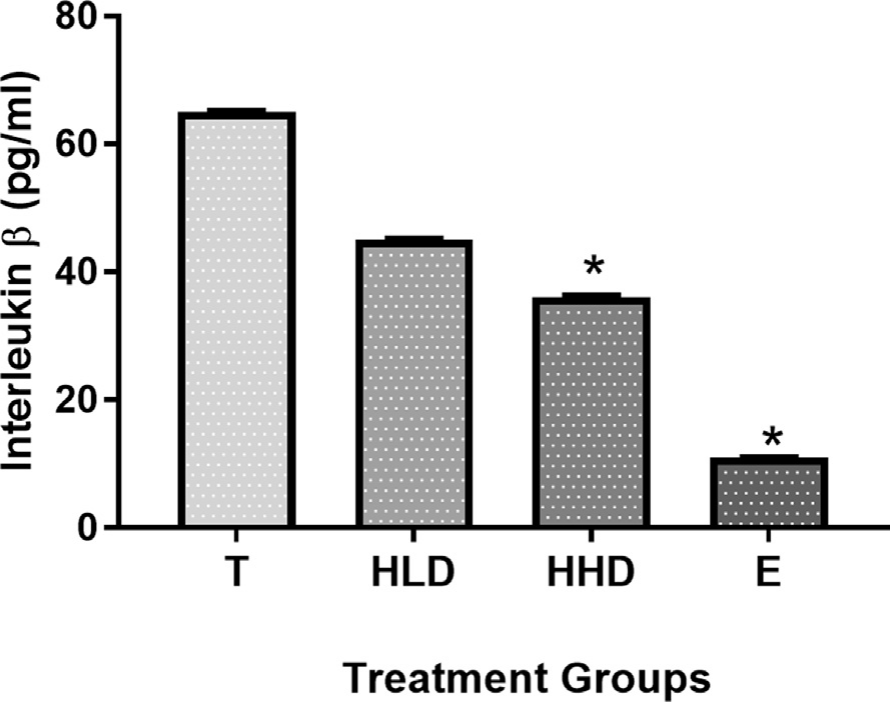
The gastroprotective effect of *Hypericum perforatum* administration on rat's blood IL-1β level. T: (10% Tween 20), HLD: (100 mg/kg *Hypericum perforatum*), HHD: (200 mg/kg *Hypericum perforatum*) and O: (20 mg/kg Esomeprazole). (∗) Indicates significance at p < 0.05 compared to the T group.

**Figure 5:**
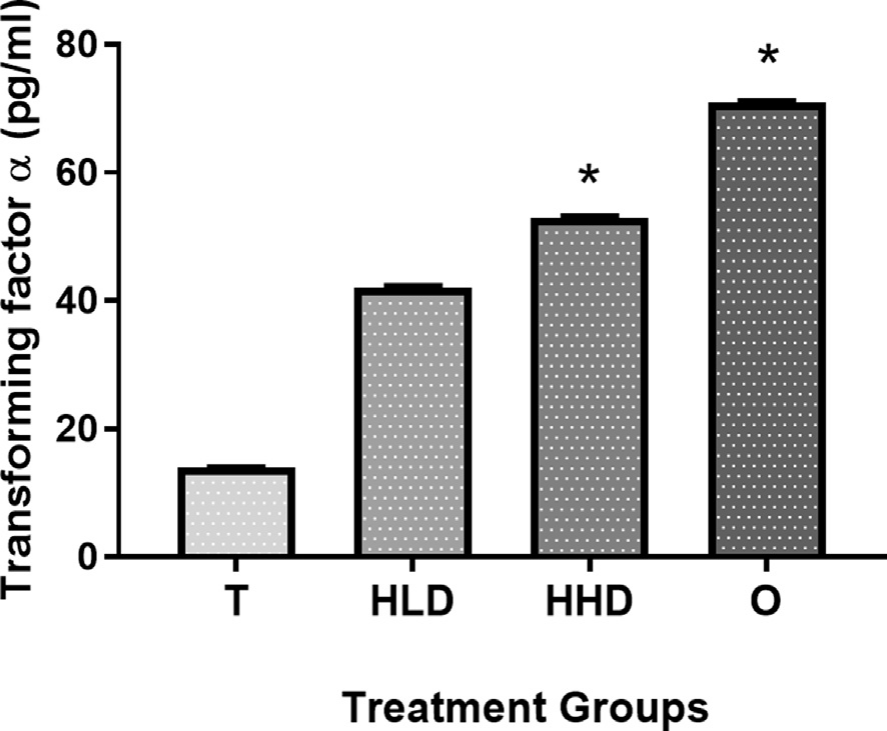
The gastroprotective effect of *Hypericum perforatum* administration on rat's blood TNFα level. T: (10% Tween 20), HLD: (100 mg/kg *Hypericum perforatum*), HHD: (200 mg/kg *Hypericum perforatum*) and O: (20 mg/kg Esomeprazole). (∗) Indicates significance at p < 0.05 compared to the T group.

Results of IL-1β and TNFα cytokines analysis showed that administration of both doses of *Hypericum perforatum* decreased the level of IL-1β and increased the level of TNFα cytokines in comparison to the ulcer control group (10% Tween 20). As shown in Figures 4 and 5 respectively.

## 4 Discussion

Gastric ulcer disease is characterized by the difference between gastric invasive factors like pepsin secretion, acid, nitric oxide, and lipid per-oxidation, and defensive mucosal factors like mucosal cell shedding, glycoprotein, mucin secretion and proliferation (Fatima et al., 2019). Ulcers caused by chemical inducers like ethanol and aspirin causes irritations and injury with bleeding to the gastrointestinal mucosa which are due to several causative factors, which include platelet thrombi, effects on mucosal blood flow, release of arachidonate metabolites, leukotriene, and platelet activating factor and damage to capillary endothelium (Rachchh and Jain, 2008).

The results revealed that ethanol ingestion upregulated the inflammatory response as demonstrated by increase of gastric proinflammatory TNF-α with a decline of the anti-inflammatory IL-1β. These findings are similar to the previous studies (W. Li et al., 2013; Mei et al., 2012). TNF-α has been strongly interrelated with gastric inflammation through recruitment and activation of immune cells, TNF-α also suppresses gastric microcirculation around ulcerated mucosa and thus delays its healing (Hasgul et al., 2014). On the contrary, IL-1β has been reported to down-regulate Major Histocompatibilty comples class II antigen presentation and consequent release of pro-inflammatory cytokines and thus, its diminished levels intensify gastric lesions stimulatingly (Liu et al., 2012).

The data of the current study suggest significant antiulcer activity of *Hypericum perforatum* leaf extract against ethanol induced gastric ulcers in rats. As presented by upregulation of the pro-inflammatory TNFα and downregulation of IL-1β levels. These results agrees with the results of Cayci and Dayioglu (Cayci and Dayioglu, 2009) who proved that *Hypericum perforatum* extracts healed gastric lesions induced by Hypothermic restraint stress in Wistar rats. The mechanism of the gastroprotective activity may be attributed to reduction in vascular permeability and strengthening of mucosal barrier. In addition, the presence of phytoconstituents in this plant like flavone and Quercetrin might be responsible for these actions since these constituents have been reported to enclose gastroprotective property (Coşkun et al., 2004; La Casa, Villegas, De La Lastra, Motilva and Calero, 2000; Mohod et al., 2016).

On the other hand, *Hypericum perforatum* showed a remarkable antibacterial activity which may also suggest the cytoprotective role of this plant against gastric ulcer as reported in early studies in which stress ulcer was prevented by antibacterial cytoprotective agents (Daschner et al., 1988). These results agrees with other teams works who reported that extract of *Hypericum perforatum* possess significant antibacterial activity due to higher concentration of bioactive phytoconstituents like alkaloids, phenolics, flavanoids and tanins (Nawchoo, 2012). The present study agrees with Yousuf et al. (Nawchoo, 2012) that Gram positive bacterial strains were found to be more sensitive than Gram negative bacterial strains, It may be due to the absence of lipopolysaccharide layer in Gram positive bacteria that might function as a barrier to the phytochemical substances that are responsible for antibacterial activity. On the base of the anti-biofilm activity against *Staphylococcus aureus* the results showed the ability of *Hypericum perforatum* to inhibit biofilm production of *Staphylococcus aureus,* this is due to as Schiavone et al. (2013) proposed that the stable form of hyperforin sis responsible of the inhibition of the growth and biofilm formation of *Staphylococcus aureus*.

The interactions with residues in the H+/K+ ATPase α protein active site and the plant extracts examined in docking study. In these interactions the most important requirements for docking protocol were: the proper orientations and correct conformations between the H+/K+ ATPase α protein binding site and studied compounds. Due that, best interactions with optimal Autodock Vina scores were used as measurement to understand the best conformation output among the 9 studied plant extracts. Docking results generated by AutoDock Vina program also aided in understanding potential docking interactions of inhibitors with H+/K+ ATPase α protein. Among the top-ranked poses between the studied molecules and binding sites Amentoflavone gives lowest energy, which was -10.5 kcal/mol and due to that it found to be the best H+/K+ ATPase α protein inhibitor among the studied plant extracts, followed by Quercitrin which gave -9.5 kcal/mol, thus it found to be the second best inhibitor for H+/K+ ATPase α protein.

Studies revealed that effective proton–potassium ATPase inhibitors are potential anti-ulcerative agents, since they interfere with the cascade of events of gastric ulcerations and proton blockers act at the initial step of ulcer pathogenicity, subsequent steps of ulcerations can also be inhibited (Jayaram and Dharmesh, 2014). The H^+^/K^+^ ATPase is the dimeric enzyme responsible for H^+^ secretion by the gastric parietal cells. It has been shown that Omeprazole can inhibit the α-subunit of H^+^/K^+^-ATPase covalently and imbalance the stimulated morphology of the parietal cell (Tari et al., 1991). Proton pump inhibitors are very effective in eliminating acids produce in the gastric area because it is the finishing path of acid stimulation. Parietal cells indeed are enriched with mitochondria and ingest more of ATP which produces inorganic phosphate that act as indirect extent of proton pump (H+/K+ - ATPase) activity in the formation and transport of H+ for gastric acid formation (Onasanwo et al., 2010).

Our results suggest an inhibitory potential of *Hypericum perforatum* on the proton pump activities could be associated with the interaction of enzymes at the ATP sites. The phytochemical screening of the leaf extract of *Hypericum perforatum* showed the occurrence of alkaloids, flavonoids, tannins, phenols, steriods and saponins, amongst other secondary metabolites. One of these constituents may be responsible for the gastroprotective anti-ulcer activities also other components isolated from the roots of *Hypericum* spp *were* reported to possess antiulcer activity *such as* xanthones including: hypericorin C, hypericorin D and 3,4-dihydroxy-5-methoxyxanthone (Ali et al., 2014). Additinaly, seven phenolic constituents (hypericin, pseudohypericin, rutin, hyperforin, quercitrin, hyperoside, and quercetin) were identified within in 17 wild-growing species of *Hypericum(**Smelcerovic* et al.*, 2006**)* which documented to possess wound healing and antiulcer activities.

## 5 Conclusion

The current study highlights evidences for the protective effects of *Hypericum perforatum* in a rat model of ethanol-induced gastric ulcer. These favorable actions were confirmed by ulcer measurements, suppression of gastric inflammation which was are similar to those exerted by the reference antiulcer drug Esomeprazole. *Hypericum perforatum* exhibited a proton pump inhibition and its anti-ulcer properties could be attributed to its phytochemical consistuents. Among the studied plant extracts as H+/K+ ATPase α protein inhibitor, Amentoflavone and Quercitrin have excellent binding interactions with H+/K+ ATPase α protein. Further laboratory work on the separate phytochemical constituents will suggest the actual compound responsible for its anti-ulcer properties, especially, its proton pump inhibitory activity.

### 5.1 Future research

Further studies will be carried out to check the effects of all compounds recognized from *Hypericum perforatum* including {Adhyperforin, Amentoflavone, Hyperforin, Hypericin, Hyperoside (Hyperin), Pseudohypericin, Quercetin, Quercitrin, Rutin} on enzymatic activity of gastric H,K-ATPase.

## Declarations

### Author contribution statement

Sargul H. Sofi, Sheila M. Nuraddin, Hazem A. Al-Bustany, Marwan Q. Nadir: Performed the experiments; Contributed reagents, materials, analysis tools or data.

Zahra A. Amin: Conceived and designed the experiments; Analyzed and interpreted the data; Wrote the paper.

### Funding statement

This research did not receive any specific grant from funding agencies in the public, commercial, or not-for-profit sectors.

### Competing interest statement

The authors declare no conflict of interest.

### Additional information

No additional information is available for this paper.
